# Comparative Analysis of CpG Islands among HBV Genotypes

**DOI:** 10.1371/journal.pone.0056711

**Published:** 2013-02-22

**Authors:** Yongmei Zhang, Chenxiao Li, Yijun Zhang, Haoxiang Zhu, Yaoyue Kang, Hongyan Liu, Jinyu Wang, Yanli Qin, Richeng Mao, Yi Xie, Yuxian Huang, Jiming Zhang

**Affiliations:** 1 Department of Infectious Diseases, Huashan Hospital, Shanghai Medical College, Fudan University, Shanghai, China; 2 Key Laboratory of Medical Molecular Virology (MOH & MOE), Shanghai Medical College, Fudan University, Shanghai, China; 3 Department of Basic Medical Sciences, Shanghai Medical College, Fudan University, Shanghai, China; University of Cincinnati College of Medicine, United States of America

## Abstract

DNA methylation is being increasingly recognized to play a role in regulation of hepatitis B virus (HBV) gene expression. The aim of this study was to compare the CpG island distribution among different HBV genotypes. We analyzed 176 full-length HBV genomic sequences obtained from the GenBank database, belonging to genotypes A through J, to identify the CpG islands in the HBV genomes. Our results showed that while 79 out of 176 sequences contained three conventional CpG islands (I–III) as previously described, 83 HBV sequences harbored only two of the three known islands. Novel CpG islands were identified in the remaining 14 HBV isolates and named as CpG island IV, V, and VI. Among the eight known HBV genotypes and two putative genotypes, while HBV genomes containing three CpG islands were predominant in genotypes A, B, D, E, and I; genotypes C, F, G, and H tended to contain only two CpG islands (II and III). In conclusion, the CpG islands, which are potential targets for DNA methylation mediated by the host functions, differ among HBV genotypes, and these genotype-specific differences in CpG island distribution could provide new insights into the understanding of epigenetic regulation of HBV gene expression and hepatitis B disease outcome.

## Introduction

Hepatitis B virus (HBV) causes chronic infections in more than 350 million people worldwide, resulting in liver diseases ranging from chronic hepatitis to hepatocellular carcinoma (HCC) [1]. HBV belongs to the Hepadnaviridae family, with a genome of approximately 3.2 kb in length that encodes four overlapping open reading frames (ORFs), including the surface antigen (S), core (C), polymerase (P), and X genes [2]. To date, ten HBV genotypes (A–J) have been classified [3], [4], [5] based on an intergroup divergence of 8% or more in the complete genomic sequences, with genotype I and J still speculative. HBV genotypes show distinct geographical distributions. Genotypes A and D have a wide global distribution [6], [7], [8], [9], [10], [11], while genotypes B and C are highly prevalent in Asia [12], [13], [14], [15]. Genotypes E is confined to West and Central Africa [16], while genotype F is restricted in South and Central America [17]. Genotype G is observed in USA [18], Japan [19], and Europe [20]. Genotype H is found in Central America and the southern regions of the United States [21]. The two newly identified and putative genotypes, I and J, are found in East and Southeast Asia [22], [23] and Japan, respectively.

In general, DNA methylation at the promoter region leads to repression of gene expression, because the 5-methyl-cytosine interferes with the recognition and binding of the transcriptional factors, to diminish mRNA transcription [24]. These covalent modification processes are catalyzed by host DNA methyltransferases in the nucleus [25]. HBV covalently closed circular DNA (cccDNA), the template of HBV transcription, also exist in the nuclei of infected hepatocytes as a mini-chromosome [26], [27], and is epigenetically regulated by the host defense system [28], [29]. The methylation of integrated HBV DNA sequences was described more than twenty years ago [30]. Recent studies demonstrated that both integrated and episomal HBV DNA can be methylated in human tissues [31]. Studies from two groups in Asia have confirmed the methylation of HBV cccDNA (genotype B and C), which also showed that the methylation of cccDNA regulates HBV replication fitness [32], [33], [34]. Contrary to these reports, another study in France (where genotype A and D are common) contended that the HBV genome is rarely targeted for DNA methylation in liver samples from HBV-infected patients with chronic hepatitis [35]. It is worth noting that different HBV genotypes were analyzed in these studies, which raises the possibility that the degree of DNA methylation may vary among HBV genotypes. It is well-established that DNA methylation occurs only at position C5 in CpG dinucleotides in mammals [36]. As for HBV DNA replication, lack of a proofreading function of viral polymerase leads to high mutation rates [37], which would also result in a greater degree of variability in the distribution of CpG dinucleotides.

To advance the understanding of CpG-rich regions within the HBV genome, and to investigate their potential as targets for methylation-mediated gene silencing, we used computer programs to locate the CpG islands from ten viral genotypic representatives. Mapping the distribution of CpG islands in different HBV genomes allowed us to perform a comparative analysis to verify whether genetic variations exist in the potential sites where methylation would occur. Such a comparative analysis might provide important insights into the different clinicopathological and virological features in patients infected with different HBV genotypes.

## Materials and Methods

We searched the GenBank with updated database till July 16^th^, 2012 at the National Center for Biotechnology Information, using “HBV”, “complete genome”, and “genotype” as the terms for the search query. Incomplete sequences with deletion of more than 200 bp were manually excluded [38]. We retrieved 176 representative full-length HBV genome sequences of genotypes A through J, with the subgenotypes identified. In addition, one hundred and thirty nine partial HBV genome sequences were obtained for to validate conclusions made from the analysis of full-length genomes. The following background information was extracted: genome size, isolate resources, host, country, genotype, and subgenotype.

Multiple alignments of the 176 HBV sequences were conducted using the program CLUSTALX version 1.81 (The UCD Conway Institute, Dublin, Ireland). Phylogenetic trees were constructed by the unweighted pair-group method with arithmetic mean (UPGMA) with 1000 bootstrap replicates, using the Kimura 2-parameter model, with the MEGA version 5.05 (The Biodesign Institute, Tempe, USA) software. The genotype of each analyzed sequence was compared with the original data to confirm the background genotyping information. If a discrepancy existed between the original report and the phylogenetic analysis performed in the study, we searched for the updated information, and the most recent classification prevailed.

The analysis of CpG islands was carried out using the MethPrimer (http://www.urogene.org/methprimer/index1.html) and the CpG Plot (http://www.ebi.ac.uk/Tools/emboss/cpgplot/) programs, which were used to predict CpG island by examining the GC content and the ratio observed/expected in a window size. The sizes, numbers, and locations of the CpG islands within each HBV whole genome were further extracted. The CpG islands were defined based on the following criteria: (1) a GC content of ≥50%; (2) an observed-to-expected CpG dinucleotide ratio ≥0.60; and (3) a sequence window longer than 100 bp [31], [39].

## Results

### Characteristics of the Selected Sequences

The 176 HBV sequences analyzed in the study were from viral DNA isolated from patients residing in 48 different countries (Table S1). After phylogenetic analyses of the full-length sequences and the corresponding background information, the 176 sequences were divided into 10 datasets, of genotypes A to J, as follows: genotype A (n = 18, 10.2%), genotype B (n = 31, 17.6%), genotype C (n = 42, 23.9%), genotype D (n = 34, 19.3%), genotype E (n = 8, 4.5%), genotype F (n = 18, 10.2%), genotype G (n = 12, 6.8%), genotype H (n = 9, 5.1%), genotype I (n = 3, 1.7%) and genotype J (n = 1, 0.6%). The intergroup divergence was greater than 8%, except the value between genotype C and I (recombinant genotype). (Table 1).

**Table 1:** The genome size, number and mean percentage of nucleotide sequence divergence between HBV genome from genotype A to J analyzed in this study.

	Genome size(bp)	No. selectedsequences	A	B	C	D	E	F	G	H	I	J
**A**	3221	18		0.5	0.5	0.5	0.6	0.7	0.6	0.7	0.5	0.7
**B**	3215	31	8.7		0.4	0.5	0.6	0.7	0.7	0.7	0.5	0.6
**C**	3215	42	9.3	8.6		0.5	0.6	0.7	0.7	0.7	0.4	0.6
**D**	3182	34	9.2	9.9	9.9		0.5	0.7	0.7	0.7	0.6	0.7
**E**	3212	8	9.7	10.6	10.5	8.3		0.7	0.8	0.8	0.6	0.7
**F**	3215	18	13.1	12.8	12.5	13.1	13.4		0.8	0.5	0.7	0.8
**G**	3248	12	10.5	11.3	12.0	11.1	11.5	13.5		0.8	0.6	0.8
**H**	3215	9	13.7	13.3	13.2	13.5	14.6	8.5	14.2		0.7	0.8
**I**	3215	3	8.2	8.4	7.5	9.3	9.9	12.7	10.2	13.1		0.7
**J**	3182	1	11.2	10.3	10.1	11.6	11.5	13.0	11.8	13.6	10.2	

The mean percentage of nucleotide sequence divergence between HBV genotype A to J sequences is shown. The corresponding standard deviations of the mean values are shown above the diagonal and were obtained by a bootstrap procedure (1000 replicates). Analyses were conducted using the Kimura 2-parameter model. The analysis involved 176 nucleotide sequences.

### Analysis of the CpG Islands within HBV Genomes

**Figure 1:**
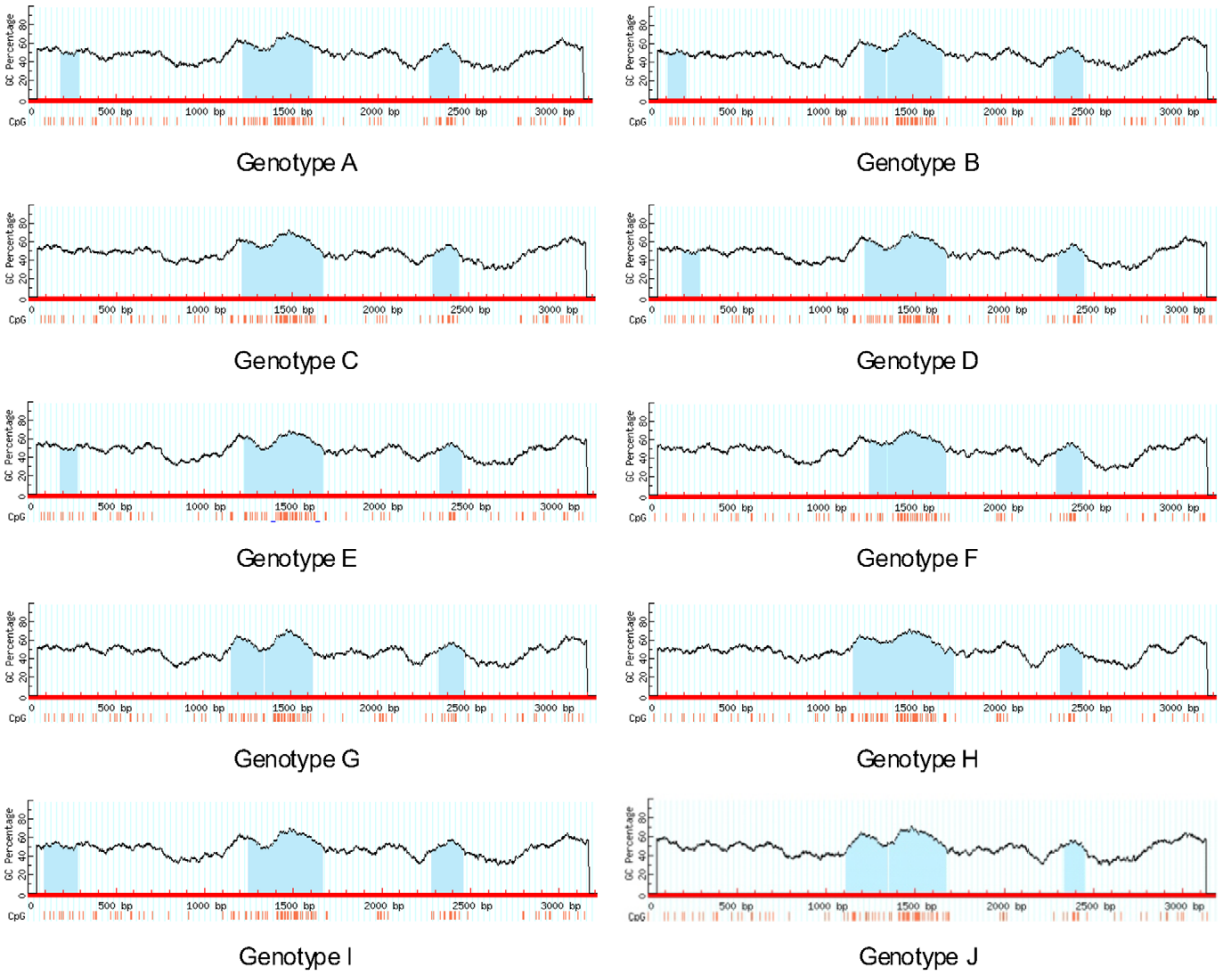
The CpG island distribution within representative HBV sequences of HBV genotypes A–J. The vertical axis refers to the GC percentage, while the horizontal axis refers to the HBV nucleotide sequence. The blue areas represent CpG islands I, II, and III. The vertical red lines indicate CpG dinucleotides.

**Figure 2:**
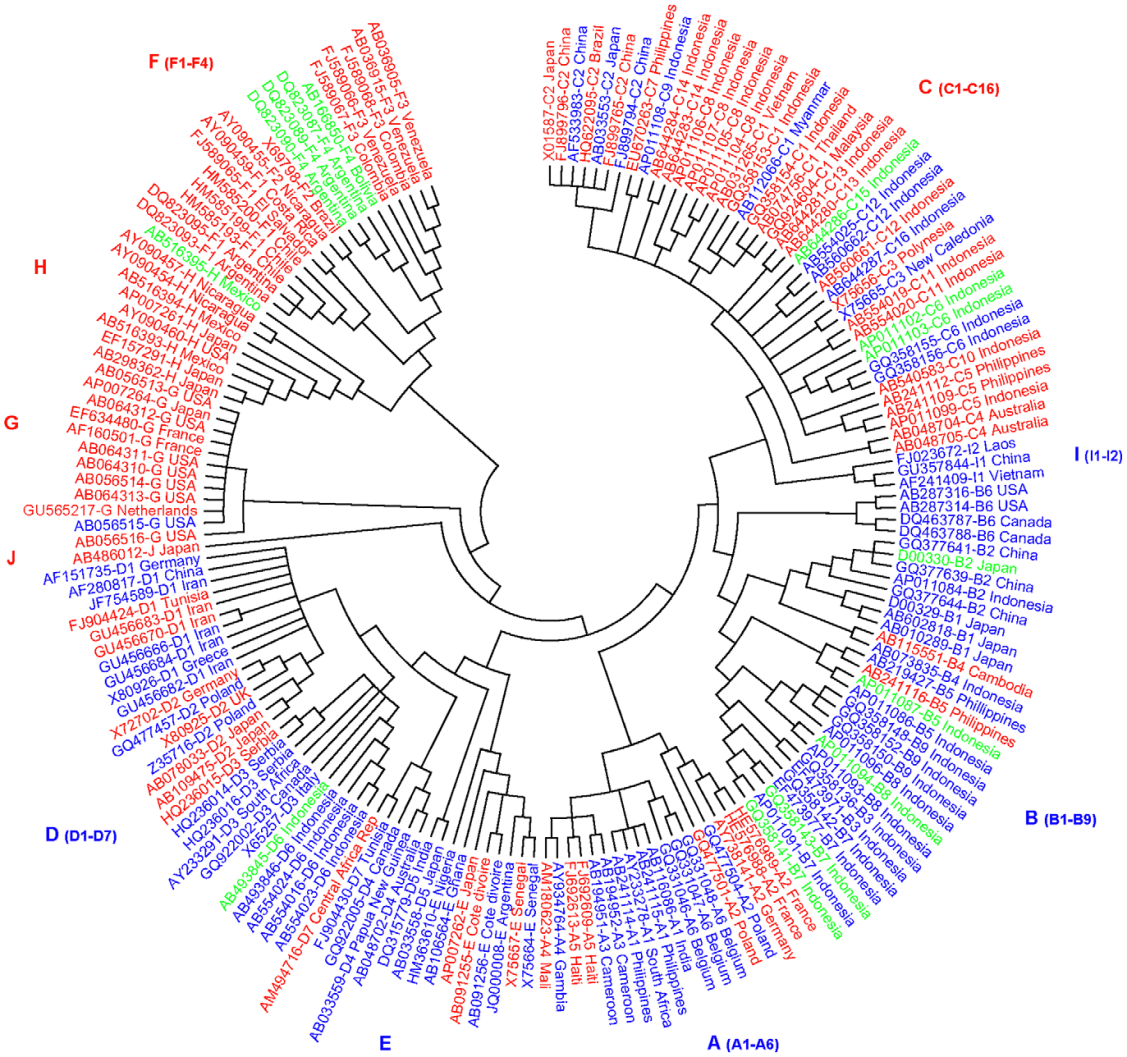
Dendrogram based on 176 complete genomic sequences of HBV with genotypes A–J. The phylogenetic tree was constructed using the UPGMA method. The genotypes are marked by the letters A through J. Each sequence is identified by the GeneBank accession number, followed by the genotype/subgenotype and the country of origin of the isolates. HBV genomes containing three, two, and four CpG islands are shown in blue, red, and green, respectively.

To comprehensively analyze the CpG islands within the HBV genomes, we mapped the islands in representative sequences of the ten HBV genotypes (Figure 1), using MethPrimer and CpG Plot to identify the location and size of CpG islands I, II, and III within each sequence. Both programs were in agreement for all CpG islands in all sequences analyzed in the study. We found that 79 of the 176 (44.9%) sequences contained three conventional CpG islands, which is in agreement with the current knowledge of CpG island distribution in the HBV genomes. However, the remaining 97 sequences contained two (n = 83, 47.2%) or four CpG islands (n = 14, 7.9%) (Figure 2). Among the ten genotypes (genotype I and J remain putative), genotypes C, F, G, and H tended to contain only two islands (CpG islands II and III), while genomes containing three CpG islands were predominant in isolates of genotypes A, B, D, E, and I. The single HBV genotype J sequence contains two islands.

### Analysis of HBV Genomes Containing the Three Conventional CpG Islands

**Figure 3:**
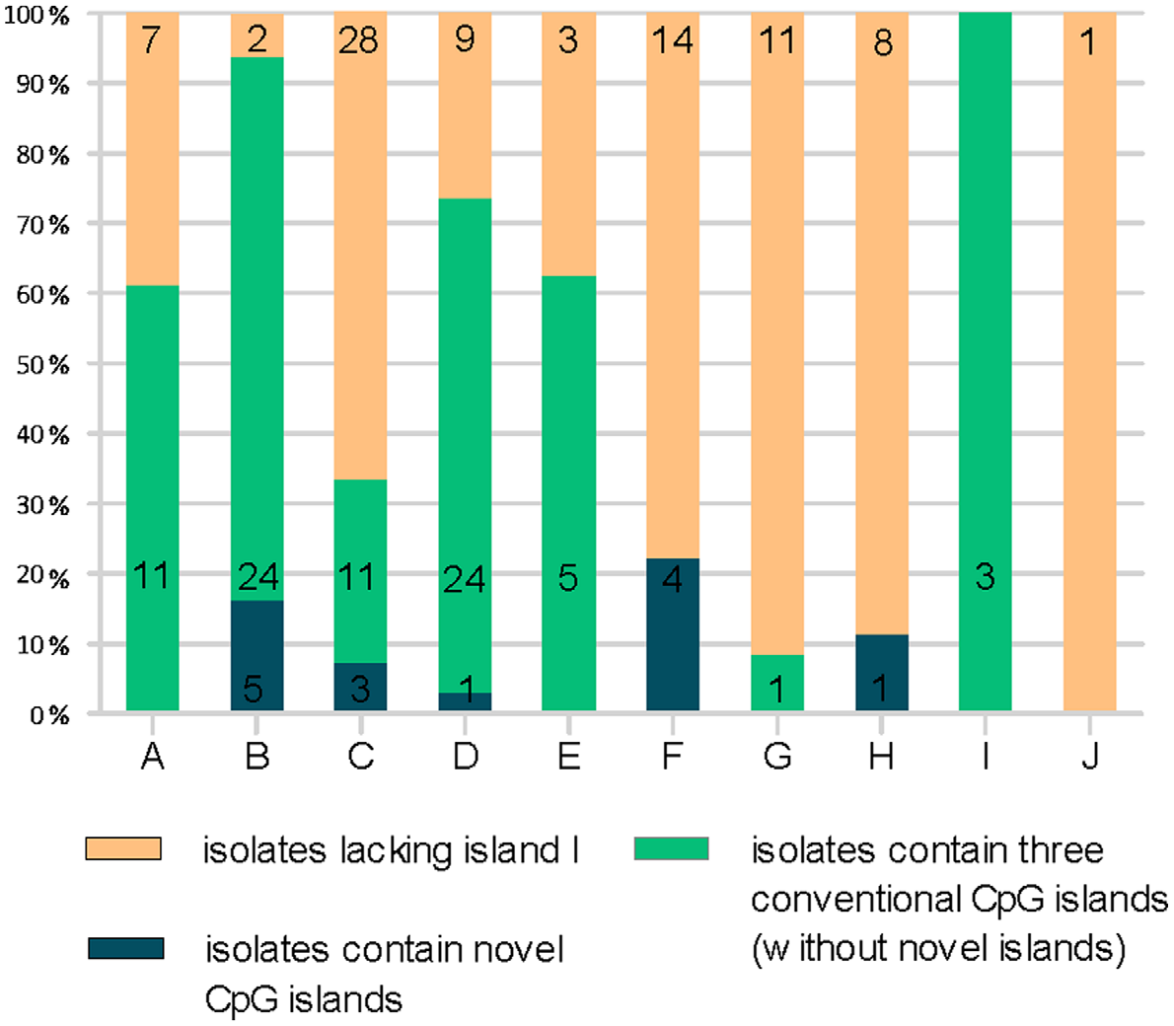
Number of HBV genomes without CpG island I, containing three conventional islands or novel islands among different genotypes.

Overall, 79 sequences contained three conventional CpG islands. The proportion of HBV genomes containing three conventional CpG islands were 61.11% (11/18) in genotype A, 77.42% (24/31) in genotype B, 26.19% (11/42) in genotype C, 70.59% (24/34) in genotype D, 62.5% (5/8) in genotype E, 0% (0/18) in genotype F, 8.33% (1/12) in genotype G, 0% (0/9) in genotype H, and 100% (3/3) in genotype I sequences, 0% (0/1) in genotype J (Figure 3). We also identified the location and the average size of the three CpG islands within the sequences of each genotype analyzed in this study. These data are summarized in Table 2. The locations of the three islands are similar among the ten genotypes despite some minor differences. CpG island I spans the start site of S gene. CpG island II overlaps the enhancer I and the X gene promoter, and CpG island III encompasses the start site of the P gene.

**Table 2:** The location and size of the three conventional and novel CpG islands within HBV sequences of different genotypes.

Genotype	CpG island I		CpG island II		CpG island III		Novel CpG islands	
	Location (bp)	Average Size (bp)	Location (bp)	Average Size (bp)	Location (bp)	Average Size (bp)	Location (bp)	Average Size (bp)
**A**	94–303	151	1215–1671	424	2276–2460	149	–	–
**B**	109–289	121	1175–1679	444	2298–2462	146	300–633 1926–2043	109
**C**	60–626	124	1035–1732	442	2121–2458	162	2874–2989	105
**D**	94–288	110	1205–1671	419	2250–2458	150	467–589	122
**E**	184–577	102	1223–1673	409	2334–2456	122	–	–
**F**	–	–	1202–1672	347	2257–2462	153	1921–2038	111
**G**	186–297	111	1163–1906	466	2341–2494	145	–	–
**H**	332–436	105	1106–1728	518	2336–2464	120	1933–2035	103
**I**	098–283	138	1248–1678	422	2252–2456	186	–	–
**J**	–	–	1111–1671	561	2335–2446	112	–	–

–, CpG island was absent. The first T of the *Eco*RI cleavage site is position 1. The numbering of the nucleotides was determined using an alignment of representative sequences of HBVs of the ten genotypes obtained from GenBank.

### Analysis of HBV Genomes Lacking CpG Island I

In total, approximately 50% (88/176) of the HBV sequences analyzed lacked CpG island I. For some genotypes, especially genotypes C, F, G, and H, the G+C content in the first CpG-rich region did not reach 50%. Alternatively, the sequence window was shorter than 100 bp, which resulted in the loss of CpG island I. Among these, 83 contained just CpG island II and III, while the remaining five sequences (AP011102, DQ823087, DQ823089, DQ823090, and AB166850) contained a novel CpG island. Two CpG islands were present in 38.89% (7/18) of genotype A sequences, 6.45% (2/31) of genotype B sequences, 66.67% (28/42) of genotype C sequences, 26.47% (9/34) of genotype D sequences, 37.5% (3/8) of genotype E sequences, 77.78% (14/18) of genotype F sequences, 91.67% (11/12) of genotype G sequences, 88.89% (8/9) of genotype H sequences, 0% (0/3) of genotype I sequences, and of the single genotype J sequences (Figure 3). The detailed information in each subgenotype was shown in Figure S1. The percentage of HBV genomes lacking CpG island I was comparable in the 139 partial genome sequences analyzed (Table S2, Figure S2).

### Novel CpG Islands within the HBV Genomes

**Figure 4:**
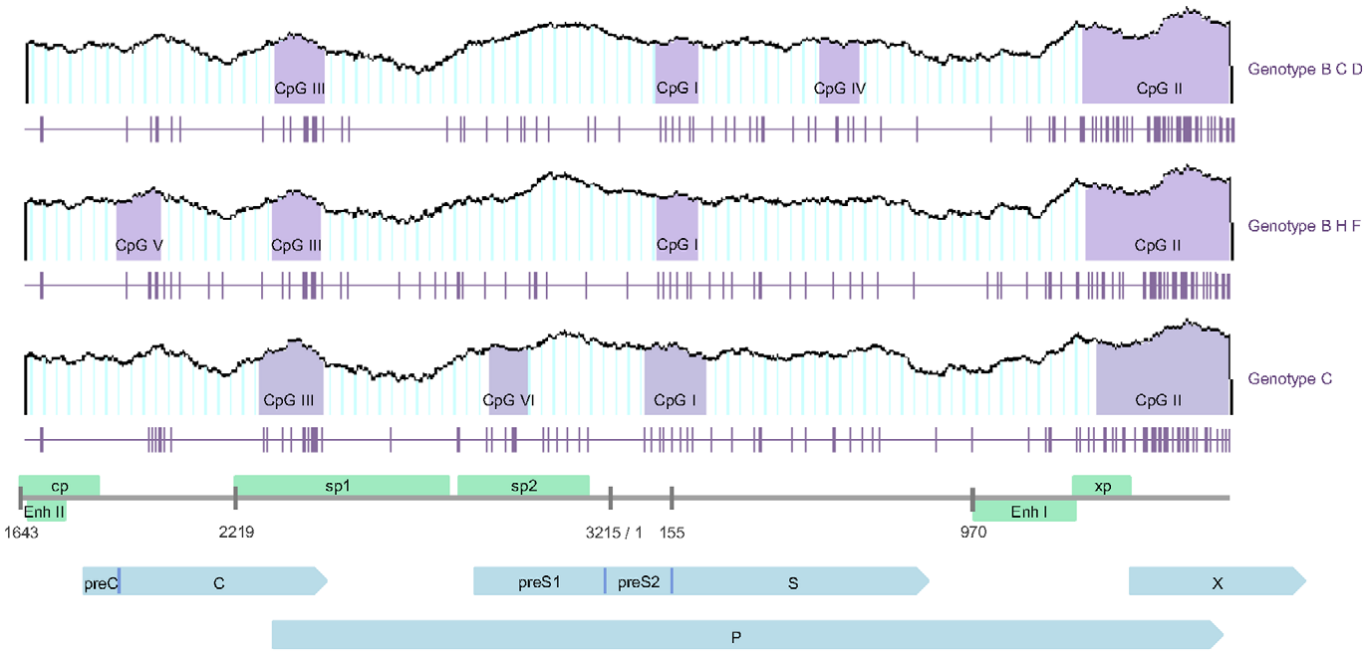
The three novel CpG islands identified in the HBV genome. The open reading frames of the pre-core/core, polymerase, surface antigen, and X genes are indicated as blue arrows. The four promoters, cp, sp1, sp2, and xp, and main regulatory elements, enhancers I and II (EnhI and EnhII), are indicated as green boxes. The purple areas represent CpG islands I to VI within the HBV genome. Each vertical purple line below indicates a single CpG dinucleotide. The nucleotide position is labeled according to the consensus sequence of HBV genomes with genotype C (EMBL: Y18855–Y18858).

We identified fourteen HBV sequences containing novel CpG islands, which were located in three different regions. The novel CpG islands were found in 16.13% (5/31) of genotype B sequences, 7.14% (3/42) of genotype C sequences, 2.94% (1/34) of genotype D sequences, 22.22% (4/18) of genotype F sequences, 11.11% (1/9) of genotype H sequences (Figure 3). Island IV was identified in six strains, AP011087, GQ358141, GQ358143, AP011094 (all genotype B); AB644286 (genotype C); and AB493845 (genotype D), all isolated from Indonesia, This island was identified in a region downstream of CpG island I, overlapping with the S gene. Island V was found in the following six strains, D00330 (genotype B) from Japan, DQ823087, DQ823089, DQ823090, AB166850 (all genotype F) from Argentina or Bolivia, and AB516395 (genotype H) from Mexico. Island V is located upstream of CpG island III, close to enhancer II and the core gene promoter, and also overlapped with C gene. Lastly, island VI was present in strain AP011102 and AP011103 (genotype C) from Indonesia. It is downstream of CpG island III and spans the sp2 promoter and the preS2 gene (Figure 4).

## Discussion

The HBV genome, in the cccDNA form of the viral mini-chromosome, is epigenetically regulated in the nuclei of infected hepatocytes 33. A numbers of studies have focused on HBV DNA methylation and histone acetylation, which have been implicated in the silencing of transcription and down-regulation of viral replication [28], [32], [34], [40]. Since DNA methylation is CpG site-specific in mammals [36], the density and location of CpG dinucleotides could have a direct impact on the methylation of the HBV genome. Our study found that there is a difference in the distribution of CpG islands among different HBV genotypes. These data provide new insights into the epigenetic regulation of the HBV genome.

As previously reported, majority of HBV isolates contain three CpG-rich regions overlapping the start site of the S gene (island I), the region encompassing enhancer I and the X gene promoter (island II), and the Sp1 promoter and the start codon of the P gene (island III) [31], [35]. Island II has been reported to be a candidate for regulation of surface gene expression, since the methylation level of the island is higher in samples with low level or no HBsAg production [41]. In addition, island II is in close proximity to core gene promoter and enhancer II. It is speculated that hypermethylation of island II can suppress transcription of Pre-C/C gene, and consequently reduced HBeAg expression [32]. What’s more, the lack of proofreading activity of the *HBV* reverse transcriptase, which accounts for a high mutation frequency 37, together with the high rate of replication may have led to difference in the density of CpG dinucleotides throughout the HBV genome. Our results showed that approximately 50% of HBV sequences analyzed contained only two islands, because the density of CpG dinucleotides within the first CpG-rich region was too low. The distribution of CpG islands appears different among HBV genotypes, which raises the possibility that DNA methylation may regulate HBV transcription to varying degrees in different genotypes. This finding might explain the contradictory results from various studies that examined the methylation of HBV DNA, since the studies were conducted on different genotypes of the HBV, and the low CpG dinucleotide density within island I may account for the limited degree of genomic methylation. However, the function of CpG island I remains unclear, and further studies are needed to verify the hypothesis that the limited genomic methylation seen in some studies is a direct result of the low CpG density of island I. In contrast to island I, islands II and III were more conserved across genotypes, and were observed in all the 176 HBV sequences analyzed.

Our study describes three novel CpG islands for the first time, which we named CpG island IV, V, VI. These islands could be potential novel targets for DNA methylation. Island IV (genotype B, C and D) is located between island I and II, the region overlapping the S and P gene. Island V (genotype B, H and F) is mapped downstream to enhancer II and core promoter, and upstream to sp1. It overlaps with C gene. Since island V is adjacent to the start site of C gene, its hypermethylation may block the translation initiation of C. Island VI (genotype C) is situated in sp2, and overlap with preS1 and P gene. Based on the position, hypermethylation of island VI may suppress transcription of 2.1 kb mRNA [42]. Nevertheless, the roles of these newly identified hypothetic CpG islands are unknown and further investigations are thus required.

In 2006, a nationwide epidemiological study reported that 7.18% of the population between the ages of 1 to 59 in China were hepatitis B surface antigen (HBsAg) carriers, and that 93 million people had chronic HBV infections [43]. Two major HBV genotypes (B and C) are prevalent in China [43], [44], [45]. It is known that heterogeneity in clinical manifestation exists between infections by these two HBV genotypes. Available data indicate that infection with genotype B is associated with earlier hepatitis B e antigen (HBeAg) seroconversion, and genotype C is associated with a higher risk of developing cirrhosis and HCC, in comparison to genotype B [46], [47]. However, the HBV-genotype specific mechanisms that contribute to these differences remain unclear. Our results showed that the CpG dinucleotide denity is relatively low in genotype C, and the absence of the CpG island I is more frequent in genotype C (69.0%), than genotype B (6.5%). We therefore speculate that the first CpG-rich region of genotype C is less likely to be targeted for DNA methylation, which may be associated with the clinical differences between the two genotypes.

In summary, our results suggest that heterogeneity exists in the CpG sites distribution within HBV sequences of different genotypes. Genotypes C, F, G, and H HBV sequences tend to contain two CpG islands, while most strains of genotypes A, B, D, E, and I contain three CpG islands, as previously reported. Moreover, three novel CpG islands were identified for the first time, as additional potential targets for DNA methylation. The different CpG island distributions among different HBV genotypes may play a role in altering the clinical outcomes of HBV infection by differential regulation of gene expression. Further studies are needed to confirm our CpG island prediction and to determine whether the HBV genotype specificity play a role in the DNA methylation-mediated epigenetic regulation of the HBV expression and replication.

## Supporting Information

Figure S1 **Number of HBV genomes with or without CpG island I within different subgenotypes.**
(TIFF)

Figure S2 **Number of HBV genomes with or without CpG island I within 139 partial genome sequences belonging to different genotypes.**
(TIF)

Table S1 **The genotypes/subgenotypes, countries of origin of isolates, and GenBank accession numbers of the HBV sequences analyzed in this study.**
(DOC)

Table S2 **The genotypes, countries of origin of isolates, and GenBank accession numbers of the HBV sequences (partial) analyzed in this study.**
(DOCX)
