# Supplementary figures and images for: Comparative Analysis of CpG Islands among HBV Genotypes
Source: PLoS One. 2013 Feb 22;8(2):e56711. doi: 10.1371/journal.pone.0056711 (PMC3579858; doi:10.1371/journal.pone.0056711)

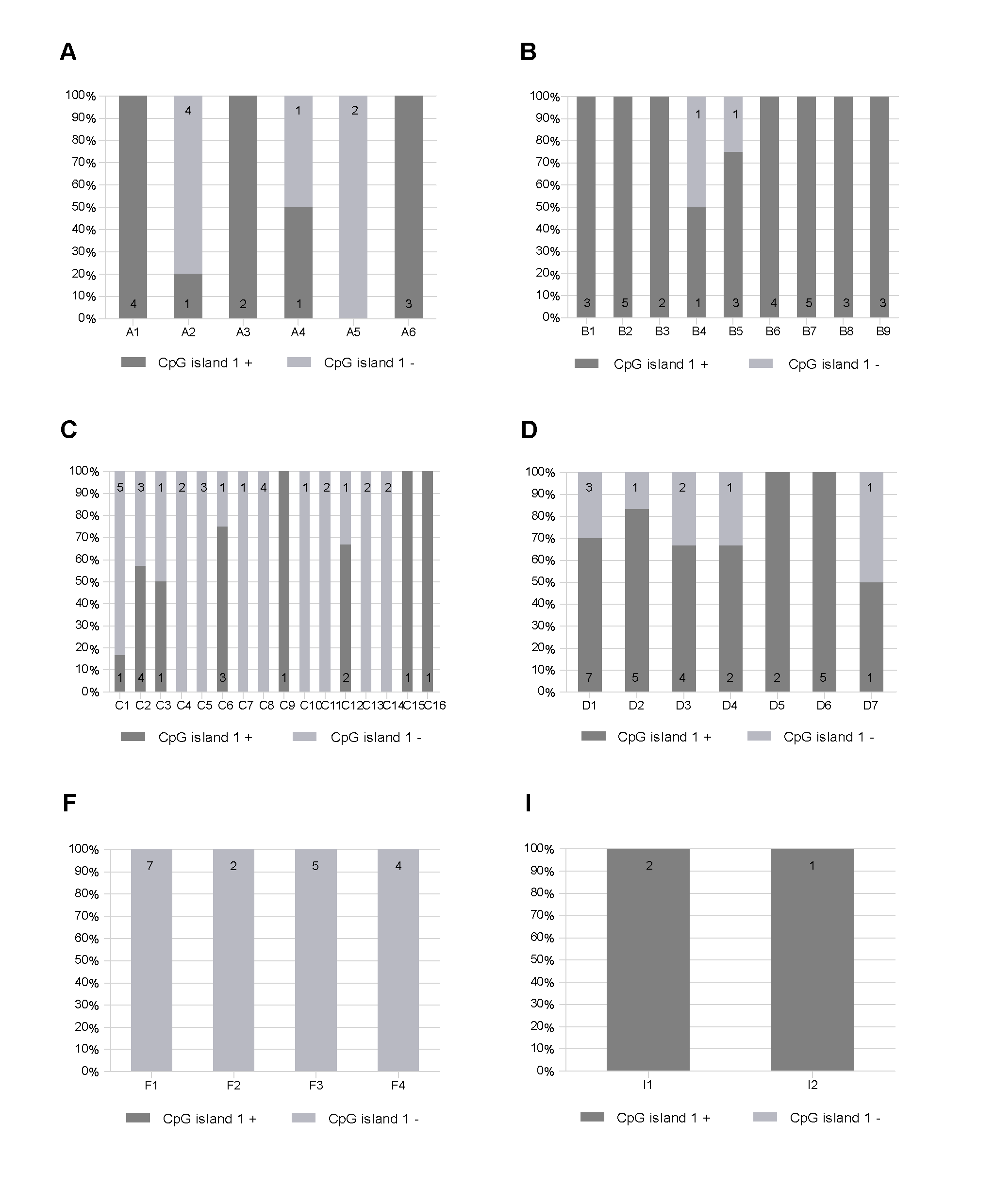

Supplement: Figure S1 — Number of HBV genomes with or without CpG island I within different subgenotypes. (TIFF) [file pone.0056711.s001.tiff]

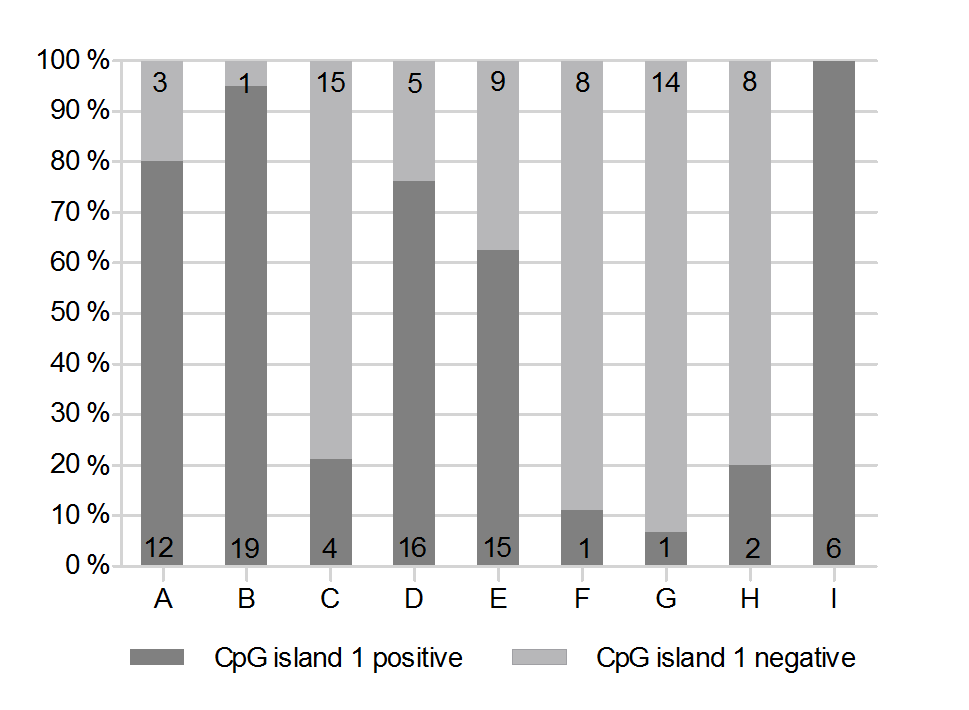

Supplement: Figure S2 — Number of HBV genomes with or without CpG island I within 139 partial genome sequences belonging to different genotypes. (TIF) [file pone.0056711.s002.tif]
