# Supplementary material for: Comparative Analysis of CpG Islands among HBV Genotypes
Source: PLoS One. 2013 Feb 22;8(2):e56711. doi: 10.1371/journal.pone.0056711 (PMC3579858; doi:10.1371/journal.pone.0056711)
Supplement: Table S1 — The genotypes/subgenotypes, countries of origin of isolates, and GenBank accession numbers of the HBV sequences analyzed in this study. (DOC) [file pone.0056711.s003.doc]

**Table S**1. The genotypes/subgenotypes, countries of origin of isolates, and GenBank accession numbers of the HBV sequences analyzed in this study

| **Genotype** | **Subgenotype** | **Country** | **GenBank Accession number** |
| --- | --- | --- | --- |
| **A** | A1 | South Africa, Philippines India | AY233278, AB241114, AB241115, AB116086 |
|  | A2 | France, Poland, Germany | HE576988, HE576989, GQ477501, GQ477504, AY738141 |
|  | A3 | Cameroon | AB194951, AB194952 |
|  | A4 | Gambia, Mali | AY934764, AM180623 |
|  | A5 | Haiti | FJ692609, FJ692613 |
|  | A6 | Belgium | GQ331046, GQ331047, GQ331048 |
| **B** | B1 | Japan | AB602818, AB010289, D00329 |
|  | B2 | Japan, Indonesia, China | D00330, AP011084, GQ377639, GQ377641, GQ377644 |
|  | B3 | Indonesia | GQ358136, EF473971 |
|  | B4 | Cambodia, Indonesia | AB115551, AB073835 |
|  | B5 | Philippines, Indonesia | AB219427, AB241116, AP011086, AP011087 |
|  | B6 | USA, Canada | AB287314, AB287316, DQ463787, DQ463788 |
|  | B7 | Indonesia | EF473977, AP011091, GQ358141, GQ358142, GQ358143 |
|  | B8 | Indonesia | AP011093, AP011094, AP011096 |
|  | B9 | Indonesia | GQ358148, GQ358150, GQ358152 |
| **C** | C1 | Vietnam, Myanmar, Malaysia, Indonesia, Thailand | AB031265, AB112066, GQ924604, GQ358153, GQ358154, AB074756 |
|  | C2 | Japan, China, Brazil | X01587, AB033553, AF533983, FJ899794, FJ899795, FJ899796, HQ622095 |
|  | C3 | Polynesia, New Caledonia | X75656, X75665 |
|  | C4 | Australia | AB048704, AB048705 |
|  | C5 | Philippines, Indonesia | AB241109, AB241110, AB241111, AB241112, AP011099 |
|  | C6 | Indonesia | AP011102, AP011103, GQ358155, GQ358156 |
|  | C7 | Philippines | EU670263 |
|  | C8 | Indonesia | AP011104, AP011105, AP011106, AP011107 |
|  | C9 | Indonesia | AP011108 |
|  | C10 | Indonesia | AB540583 |
|  | C11 | Indonesia | AB554019, AB554020 |
|  | C12 | Indonesia | AB554025, AB560661, AB560662 |
|  | C13 | Indonesia | AB644280, AB644281 |
|  | C14 | Indonesia | AB644283, AB644284 |
|  | C15 | Indonesia | AB644286 |
|  | C16 | Indonesia | AB644287 |
| **D** | D1 | Iran, Turkey, Tunisia, Greece, Germany, China | GU456682, GU456683, GU456684, GU456666, GU456670, JF754589, FJ904424, X80926, AF151735, AF280817 |
|  | D2 | Japan, Poland, UK, Germany | AB109475, AB078033, Z35716, GQ477457, X80925, X72702 |
|  | D3 | South Africa, Italy, Serbia, Canada | AY233291, X65257, HQ236014, HQ236015, HQ236016, GQ922002 |
|  | D4 | Australia, Canada, Papua New Guinea | AB048702, GQ922005, AB033559 |
|  | D5 | Japan, India | AB033558, DQ315779 |
|  | D6 | Indonesia | AB493845, AB493846, AB554016, AB554023, AB554024 |
|  | D7 | Tunisia, Central African Republic | FJ904430, AM494716 |
| **E** | E | Japan, Argentina, Ghana, Cote divoire, Senegal, Nigeria | AP007262, JQ000008, AB106564, AB091255, AB091256, X75657, X75664, HM363610 |
| **F** | F1 | El Salvador, Argentina, Costa Rica, Chile | FJ589065, DQ823093, DQ823095, AY090459, HM585193, HM585199, HM585200 |
|  | F2 | Nicaragua, Brazil | AY090455, X69798 |
|  | F3 | Colombia, Venezuela | FJ589067, FJ589068, FJ589066, AB036905, AB036915 |
|  | F4 | Argentina, Bolivia | DQ823087, DQ823089, DQ823090, AB166850 |
| **G** | G | France, USA, Netherlands, Japan | EF634480, AF160501, AB064310, AB064311, AB064312, AB064313, AB056513, AB056514, AB056515, AB056516, GU565217, AP007264 |
| **H** | H | USA, Nicaragua, Mexico, Japan | AY090460, AY090454, AY090457, AB516393, AB516394, AB516395, AP007261, AB298362, EF157291 |
| **I** | I1 | China, Vietnam | GU357844, AF241409 |
|  | I2 | Laos | FJ023672 |
| **J** | J | Japan | AB486012 |
