# Supplementary material for: Comparative Analysis of CpG Islands among HBV Genotypes
Source: PLoS One. 2013 Feb 22;8(2):e56711. doi: 10.1371/journal.pone.0056711 (PMC3579858; doi:10.1371/journal.pone.0056711)
Supplement: Table S2 — The genotypes, countries of origin of isolates, and GenBank accession numbers of the HBV sequences (partial) analyzed in this study. (DOCX) [file pone.0056711.s004.docx]

**Table S2. The genotypes, countries of origin of isolates, and GenBank accession numbers of the HBV sequences (partial) analyzed in this study**

| **Genotype** | **Country** | **GenBank Accession number** |
| --- | --- | --- |
| **A** | Japan, South Africa, France, Canada, Argentina, Malawi, Martinique, Belgium | AP007263, AY233288, AY233284, AB014370, AJ344115, AJ309370, AY128092, EU185789, AB076678, AB076679, AY233275, AY233281, U87746, HE974381, GU563558 |
| **B** | China, Indonesia, Malaysia | AF100309, D00331, FJ518812, HM011504, EU139543, AB033555, AY220698, AY220704, AF282917, JF899335, JQ040147, AB555499, GQ924640, HM011476, HM011478, AB554017, AB033554, FJ899786, AB540582, EU306698 |
| **C** | China, Japan, Malaysia, Indonesia | HM750131, D16665, D50517, AB014378, AF458665, AY066028, AF461357, AF411411, JF899336, EU439009, JQ040141, HM750141, HM750133, JF436923, HM011500, AB554021, AB554019, AB554025, GQ377586 |
| **D** | Poland, France, Sweden, China, India, Belgium, Indonesia, Syria | Z35716, AJ344116, AF121239, AF280817, AF121241, HQ833465, GQ183486, FJ349214, JN664926, JN664932, HQ833469, JN040822, JN040762, JN664927, AB554016, GU357846, JN257210, JN257200, JN664930, JN664918, AB555497 |
| **E** | Martinique, Argentina, Nigeria | HE974384, HE974380, JQ000009, JQ000008, HM363579, HM363569, HM363609, HM363611, HM363581, HM363570, HM363588, HM363597, HM363589, HM363586, HM363567, HM363577, HM363600, HM363568, HM363572, HM363571, HM363575, HM363606, HM363583, HM363594 |
| **F** | El Salvador, Costa Rica, Argentina, Japan, Panama, Ireland | AY090461, AY090458, AF223963, AB116654, AB116549, HQ378247, DQ823091, DQ823092, DQ823088 |
| **G** | USA, Argentina, Brazil, Germany, France | AB064315, AB064316, AB064314, HE981175, HE981173, HE981171, HE981176, HE981174, HE981172, AF405706, EF464097, EF464098, EF634481, DQ207798, EF464099 |
| **H** | Mexico, Argentina, Thailand, Japan | HM117850, HM117851, HM066946, FJ356716, FJ356715, EU498228, AB266536, AB179747, AB275308, AB205010 |
| **I** | China, Canada, Laos | JF899337, JF899338, EU833891, FJ023664, FJ023660, AB231908 |
